# An Architecture for Performance Optimization in a Collaborative Knowledge-Based Approach for Wireless Sensor Networks

**DOI:** 10.3390/s111009136

**Published:** 2011-09-27

**Authors:** Manuel Angel Gadeo-Martos, Jose Angel Fernandez-Prieto, Joaquin Canada-Bago, Juan Ramon Velasco

**Affiliations:** 1 Telecommunications Department, University of Jaen, Alfonso X El Sabio 28, Linares, Jaen 23700, Spain; E-Mails: jan@ujaen.es (J.A.F.-P.); jcbago@ujaen.es (J.C.-B.); 2 Department of Automatic, University of Alcala, Alcala de Henares, Madrid 28801, Spain; E-Mail: juanramon.velasco@uah.es

**Keywords:** intelligent spaces, wireless sensor networks, fuzzy rule-based systems

## Abstract

Over the past few years, Intelligent Spaces (ISs) have received the attention of many Wireless Sensor Network researchers. Recently, several studies have been devoted to identify their common capacities and to set up ISs over these networks. However, little attention has been paid to integrating Fuzzy Rule-Based Systems into collaborative Wireless Sensor Networks for the purpose of implementing ISs. This work presents a distributed architecture proposal for collaborative Fuzzy Rule-Based Systems embedded in Wireless Sensor Networks, which has been designed to optimize the implementation of ISs. This architecture includes the following: (a) an optimized design for the inference engine; (b) a visual interface; (c) a module to reduce the redundancy and complexity of the knowledge bases; (d) a module to evaluate the accuracy of the new knowledge base; (e) a module to adapt the format of the rules to the structure used by the inference engine; and (f) a communications protocol. As a real-world application of this architecture and the proposed methodologies, we show an application to the problem of modeling two plagues of the olive tree: prays (olive moth, *Prays oleae* Bern.) and repilo (caused by the fungus *Spilocaea oleagina*). The results show that the architecture presented in this paper significantly decreases the consumption of resources (memory, CPU and battery) without a substantial decrease in the accuracy of the inferred values.

## 1. Introduction

Many recent studies have focused on various aspects of Intelligent Spaces (IS) [1–3]. The IS was proposed by Lee [2] as an environmental system that is able to support humans in informative and physical ways, and as a space that contains human and artificial systems. Thus, an IS could utilize computer monitors to provide information to humans, and robots could be used to provide physical services to humans as physical agents.

Over the past few years, several definitions of ISs have been proposed; it is important to acknowledge the definitions that were presented in [4,5], in which ISs are described as spaces with many embedded and networked sensors and actuators. Their essential functions are the following: (a) to observe the space using distributed sensors; (b) to extract useful information from the obtained data; and (c) to fuse the information acquired by each sensor and to share it with other devices efficiently. Taking into account these properties, several studies [6–8] have been devoted to set up ISs over WSNs.

Over the past few years, Wireless Sensor Networks (WSNs) [9,10] have received the attention of many researchers. WSNs are defined [11] as networks that are composed of a large number of sensor nodes, and they can be conceived of as small computers with extremely basic interfaces and components. Each node consists of a processing unit with limited computational capability and memory, sensors, a communication device and a limited power source, usually in the form of a battery.

WSNs provide perfect scenarios for sensor collaboration for a global purpose. In this context, the term collaborative involves communication of individual measurements among sensors to accomplish their tasks and to achieve a common goal.

Despite the wide interest in sensors and collaborative WSNs, little attention has been paid to optimizing the integration of collaborative Fuzzy Rule-Based Systems (FRBSs) into WSNs. FRBSs [12] are considered an extension of classical rule-based systems because they deal with “IF-THEN” rules whose antecedents and consequents are composed of fuzzy logic statements (fuzzy rules) [13] instead of classical logic statements. One of the main characteristics of these integrated systems is the capacity of each node to incorporate facilities for simple sensing, actuation, communication and computation; however, the full capabilities of such networks are reached only by the cooperation of all of the nodes. Because of these qualities, these networks can efficiently provide all of the services necessary to implement an IS.

To optimize the implementation of an IS by means of a WSN, this paper presents a distributed architecture proposal for collaborative FRBSs embedded in a WSN. The main contribution of the design that is presented is to make it possible to decrease the consumption of the required resources [(a) memory; (b) CPU; and (c) battery] by an FRBS embedded in a node of a WSN, without a substantial decrease in the accuracy of the inferred values.

To achieve this goal, we include the following in the proposed architecture:

- A design of the inference engine that is based on the approximate Mamdani-type [12], to achieve a high level of accuracy in complex problem domains.
- A visual interface, to facilitate the composition of the descriptive and approximate Knowledge Bases (KBs) [12] and to achieve a high degree of interpretability of the linguistic rules and additionally, a high level of accuracy.
- A module to reduce rules, which is used to decrease the redundancy and the complexity of the fuzzy rule set without a substantial decrease in their completeness and consistency.
- A module to evaluate the accuracy of a new KB that was obtained by means of the application of the rule reduction algorithm proposed.
- A module that makes possible the transformation of the base of rules that are descriptive into approximates [12], to make the most out of approximate and descriptive FRBS advantages.
- A communications protocol that is used for the distribution of an entire or partial KB among the sensor nodes, the collection of inferred data, the management of the sensor nodes, and the integration with other measurement applications.

The remainder of this paper is organized as follows. Section 2 addresses related work and motivation. Section 3 shows the modular architecture proposed to optimize the integration of collaborative FRBSs embedded in a WSN, which includes a description of the following: an inference engine design, visual interface facilities, algorithms proposed to make rule reduction in KBs and to evaluate KB performance, and the facilities that incorporate by a module of transformation of the basis of the rules and the communications protocol. Section 4 shows the experimental results that are obtained to evaluate the performances of the proposed architecture applied to the problem of modeling the two plagues of the olive tree: prays and repilo. Finally, conclusions are drawn in Section 5.

## 2. Related Work and Motivation

Lee proposed the concept of the IS as an environmental system able to support humans in informative and physical ways and to contain human and artificial systems. These human and artificial systems become clients of the IS and, simultaneously, the artificial systems become agents of the IS. Because the whole space is an intelligent system, the IS, as a spatial system, is able to easily monitor and provide services to clients. Specific tasks that cannot be achieved by the IS alone are accomplished by utilizing its clients [2].

Investigators [1] have proposed that this research field is not necessarily closely related to robotics; however, they believe that robots under ISs have many interesting features. Thus, an IS could utilize computer monitors to provide information to humans, and robots could be used to provide physical services to humans acting as physical agents. If necessary, robots as well as humans are supported by an IS. When a robot lacks the sensors to navigate around an IS, the robot is treated as a client of the IS and the information lacked is provided to the robot by the IS.

As described in [2], an IS has two roles with respect to a robot working in it. One role is the enlargement of ability, and the other role is resource sharing. Thus, an IS has a role as an extended sensor for the robot and enhances the ability of the robot to receive a request from a distant location. Further resource sharing occurs when more than one robot uses the resources of an IS, and robots can thus decrease their common resources. However, an IS does not aim to dispense with sensors or robots’ autonomy; rather, it supports a robot by providing the resources it lacks to act as a normal robot, while helping a robot with adequate resources to act as an even better robot.

Intelligent environments were described in [3] as complex systems (*i.e.*, rooms) in which humans and machines collaborate to accomplish a task. From such a perspective, intelligent environments can also be considered as a novel human-machine interface, and the overall goal of the research will be to design and develop integrated sensor-based systems that allow natural and efficient mechanisms for human-computer interactions in places where humans work, learn, relax, and play.

To have a well-accepted definition of an IS, Mohan proposed four requirements that a physical space needs to possess in order to be called intelligent:

- ISs are designed for humans, and they should facilitate normal human activities taking place in these spaces.
- ISs should automatically capture and dynamically maintain an awareness of the events and activities taking place in these spaces.
- ISs should be responsive to specific events and triggers.
- ISs should be robust and adaptive to various dynamic changes.

In [4], smart environments that are also called ISs are described as spaces with many embedded and networked sensors and actuators, where the essential functions of these smart environments are the following:

- To observe the space using distributed sensors.
- To extract useful information from the data obtained and to provide various services.

Other properties of the ISs are described in [4,5] as the following:

- The system must have a function that fuses the information acquired by each sensor and that shares it with other devices efficiently.
- The system should have flexibility and scalability.

In this proposed concept of an IS, sensor nodes are also distributed in the space because it is necessary to reduce the network load in a large-scale network, and it can be built to process raw data at each sensor node before collecting the information. To realize detailed observation over the whole space, it is necessary for networked sensors to cooperate with each other.

The field of research on ISs that are based on distributed sensor systems has been expanding recently [14], and many researchers have developed designs of systems for providing informative services to end users (e.g., support during a meeting [15], health care [16], support of the elderly [17], information display using a pan-tilt projector [18], support of human activities [19] and robot localization [20,21]).

On the other hand, WSNs are defined [9] as networks that are composed of a large number of sensor nodes and that can be conceived of as small computers with extremely basic interfaces and components. Each node consists of a processing unit with limited computational capability and memory, sensors, a communication device and a limited power source, usually in the form of a battery.

These properties of the sensor nodes of the WSNs allow them to take advantage of their process and communications capacities to implement the main characteristics of an IS, which are the following: to act as a server or client and to provide services to the clients; to fuse information; to share information and resources; to capture and dynamically maintain an awareness of the events; to be responsive to specific events and triggers; to be robust and adaptive to dynamic changes; and to have flexibility and scalability. On the other hand, the capacities of WSNs enable them to implement the distribution of a control algorithm of a rule-based system [22].

Because of these qualities, several studies have recently been devoted to set up ISs over WSNs, such as the following:

- In [6], an implementation example for setting up an IS concept is described; this method is implemented with several communications technologies, including WSNs. As indicated in [6], WSNs are the foothold of the future generation of IS systems. They serve as acquisition tools in ambient circumstances to replace or upgrade users’ experiences. Successful ambient adaptation leads to automatic execution of users’ activities and needs.
- In [7], a ZigBee WSN-based detection and help system for elderly abnormal behaviors is presented with service robots in the IS.
- In [8], a novel model of a WSN for Smart Civil Structures and Intelligent Building has been presented. This proposed model aims to make civil structures and buildings “intelligent” by deploying WSNs to provide significant improvements in the areas of fire protection, safety, security, comfort, building automation, and communications.

As has been described above, an IS implementation, at minimum, requires the system to have a function that fuses the information acquired by each sensor and shares it with other devices efficiently. However, none of the schemes presented above describes the algorithm used to perform the tasks of information fusion and sharing.

The study in [23] shows how FRBSs can control artificial and natural lighting automatically, in an intelligent residential lighting control system based on a ZigBee wireless sensor network.

To control artificial and natural lighting automatically, an intelligent residential lighting control system with a ZigBee wireless sensor network is used to collect the environmental data and transmit the data to a PC, where a fuzzy system is executed to generate the lighting control signal, which is transmitted through the WSN. The execution of these tasks requires a significant amount of resources in terms of wireless communications’ bandwidth and battery consumption.

To improve the above-mentioned problem, a choice to take into account is the use of a fuzzy rule-based collaborative and cooperative approach.

Next, the definitions of the terms collaborative and cooperative are presented:

- The term collaborative involves communication of individual measures among sensors to accomplish their tasks and to achieve a common goal [24].
- A cooperating object (CO) was defined as a single entity or a collection of entities consisting of sensors, controllers (information processors), actuators or cooperating objects that communicate with each other and are able to achieve, more or less autonomously, a common goal [25,26].

Therefore, the integration of FRBS into WSNs is typical in examples of such collaborative and cooperating objects. These networks consist of objects that are individually capable of simple sensing, actuation, communication and computation, but the full capabilities of such networks are reached only by the cooperation of all of these objects. Because of these qualities, these networks can efficiently provide all of the services necessary to implement an IS. To the best of our knowledge, two collaborative and cooperative approaches that integrate fuzzy rule-based systems into WSNs have been presented:

- The approach titled D-FLER has been presented in [27]. This system incorporates distributed and embedded collaborative mechanisms for reasoning about the observed data and making decisions or taking actions in a coordinated manner. It uses two types of inputs: individual observations (sensor readings of the current node) and neighborhood observations (fuzzified sensor data from the neighboring nodes). However, D-FLER has some limitations, derived from the need to transmit a significant amount of data to neighboring nodes and to process a significant amount of data.
- The approach presented in [11] is a new scheme that fuses the following: the individual observations and the knowledge that was obtained from the neighboring nodes. The innovation presented involves the use of a FRBS to define collaborative knowledge. This approach allows the users to define the collaboration among sensors by means of a specific KB (variables, fuzzy sets and rules), which presents significant advantages:
  1. The collaborative scheme may address uncertainty and imprecision.
  2. It is possible to separate control or modeling knowledge from collaborative knowledge, using interpretable rules in both cases.
  3. The collaborative approach may support sensor failures and communication errors because, in this case, the collaborative sensor would infer a proportional value to the number of failures between the control or modeling knowledge (local knowledge without a collaborative scheme) and the collaborative scheme.

In addition, this last proposal can achieve these mentioned advantages with a decrease in the amount of data to process and transmit.

Some scenarios that could benefit from the proposed approach are ISs that perform tasks of controlling or modeling in which sensor inferences may be affected by measures or inferences of neighboring sensors, e.g., event detections (fire detection, alarms), environmental monitoring (water quality, pest detection), industrial process control, and robotics.

However, these proposals also present some drawbacks associated with the following limitations:

- The scheme does not include a human-machine interface that makes it easy to obtain the necessary knowledge and the visualization of data.
- The scheme does not describe a FRBS specifically adapted to the sensor limitations.

To make it easy and efficient to develop and set up an IS with WSNs and embedded FRBSs, in this work we propose the use of the following:

- A fuzzy rule-based collaborative and a cooperative approach.
- A modified structure over the structure presented in [11], which includes an interface to make possible the knowledge base (KB) edition and to obtain operation results, and a FRBS designed to increase sensor performance.

On the other hand, the use of an interface makes this work easy but does not guarantee an optimal KB generator. In addition, this new scheme introduces some additional modules proposed to optimize these KBs by means of rule redundancy reductions and rule adaptations to specific modeling problems.

## 3. Distributed Architecture Proposal for a Collaborative Fuzzy Rule-Based System Embedded in a Wireless Sensor Network

A modular architecture has been proposed to implement the following tasks, which were mentioned above: KB construction, rule reduction, rule adaptation, KB transmission and obtaining the operations results.

**Figure 1.**
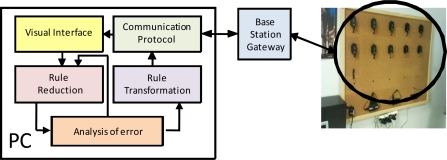
Modular architecture.

**Figure 2.**
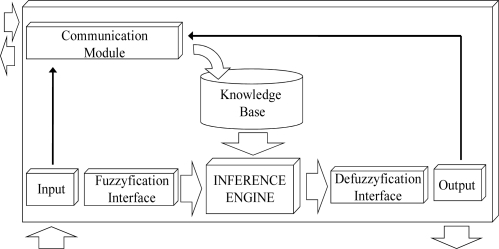
Basic structure of a Mamdani-FBRS embedded into a sensor.

This architecture (Figure 1) provides the following: (1) In the Personal Computer: (a) a visual interface for easy fuzzy rule development; (b) a module for rule reduction to optimize the generated KB; (c) a module for the analysis of error, to evaluate the accuracy reduction; (d) a module for rule transformation that facilitates the adaptation of rules to optimize the work of the FRBS inference engine; and (e) the communications protocol, which is being designed to manage multipurpose sensor networks, to transmit this KB to the desired nodes and to receive data from the sensor nodes. (2) In the sensor node: the basic elements of the FRBS (Figure 2).

### 3.1. Design of the Inference Engine

The FRBS proposed in this paper is based on the model of Mamdani [28] because it provides a highly flexible means of formulating knowledge while at the same time the knowledge remains interpretable. Two variants of Mamdani FRBSs have been proposed [12]: (1) descriptive and (2) approximate [29–32]. The structures of these systems are similar, but each type of FRBS has different properties and presents complementary advantages and drawbacks. To improve embedded FRBS behavior, it is possible to use these advantages.

In descriptive FBRS (or linguistic Mamdani FRBS), rules carry a linguistic label that points to a specific fuzzy set of a linguistic partition of the underlying linguistic variable. In approximate FBRS rules, the input variables and the output variables are fuzzy variables instead of linguistic variables.

Approximate FRBSs demonstrate some specific advantages over linguistic FRBSs, making them especially useful for certain types of applications [31]: (1) each rule employs its own distinct fuzzy sets, resulting in additional degrees of freedom and an increase in expressiveness and (2) the number of rules can be adapted to the complexity of the problem. These properties enable approximate FRBSs to achieve a better degree of accuracy than linguistic FRBS in complex problem domains. In descriptive FRBSs, the main advantage is the large degree of interpretability of the linguistic rules [33–36].

To make the most out of approximate and descriptive FRBS advantages, this approach enables the use of a visual interface to facilitate the composition of the descriptive and approximate KBs, while the inference engine of the embedded FRBS works as an approximate Mamdani-type.

To reduce the computational burden, this paper proposes the use of an FRBS in the mode B-FITA (First Infer, Then Aggregate) and with the operator center of gravity.

### 3.2. Design for the Base of Rules

As was proposed by Cordon *et al*. [12], the performance of an FRBS depends on two aspects: (a) the way in which the fuzzy inference process is developed and (b) the composition of the fuzzy rule set. In this sense, the following properties of fuzzy rule sets are beneficial and improve their approximation accuracy:

- The completeness of the fuzzy rule set. An FRBS obeys the completeness property if for each conceivable system input it infers to a corresponding output, and for an arbitrary input at least one of the fuzzy rules has to trigger.
- The consistency of the fuzzy rule set. A generic set of rules is consistent if it does not contain contradictory rules. Thus, two descriptive rules are inconsistent when they have the same antecedent and have mutually exclusive consequents.
- The low complexity of a fuzzy rule set. This property is concerned with the number of fuzzy rules composing the rule base. Low complexity becomes important in control problems, as the main requirements are usually the process speed and the simplicity of the base rather than the accuracy response, and it is equally beneficial in linguistic modeling applications because the main goal is to obtain a human-readable description of the real system.
- The redundancy of a fuzzy rule set. These If-Then rules have the property that a system state is covered by more than one rule because the fuzzy sets in the antecedents overlap. The existence of redundant rules may cause degradation in the performance of the FBRS. Therefore, it is important to take a closer look at redundancy for the purpose of removing unnecessary, detrimental rules from fuzzy rule sets.

#### 3.2.1. Visual Interface to Compose the Base of Rules

In this paper, we propose the use of a visual interface as a method of KB composition. Using this interface, the human expert can specify the linguistic labels associated with each linguistic variable, the structure of the rules in the rule base (RB), and the meaning of each label. This method is the simplest method to be applied when the expert is able to express his knowledge in the form of linguistic rules. To improve the accuracy of this linguistic approach, this interface enables the specification of exact membership functions as well. When implementing earlier recommendations, the composed KB would have the following:

- It must cover all of the possible systems space of the input variables.
- It must not have rules with the same antecedent and mutually exclusive consequents.
- It must be composed by the minor number of rules possible, to decrease the memory and CPU consumption.
- It must not have redundant rules, with the same antecedent and overlap in the fuzzy set of the consequents.

#### 3.2.2. Algorithm Proposed to Make Rule Reduction in the Base of Rules

In the approach proposed in this paper, after the KB is composed, a module to reduce rules is used, to decrease the redundancy and the complexity of the fuzzy rule set without a substantial decrease in their completeness and consistency.

Several methods have been proposed for optimizing the size of the rule base obtained with automated modeling techniques such as compatible cluster merging [37], fuzzy binary box tree [38], or membership function fusion and annihilation [39]. In [40], a fuzzy genetic algorithm is presented, and in [41], a supervised inductive algorithm is presented; each of these algorithms has a procedure (based on different criteria) that extracts from the population the minimum number of rules required to formulate the final RB. The study in [29] presents a measure of overlap that allows redundant rules to be identified, and a criterion based on this measure of overlap is then proposed to decide whether a rule should be discarded from the rule set. In other studies [42–45], a genetic process for removing redundant rules from fuzzy rule sets in classification and modeling has been proposed.

As has been defined in [46], redundant rules include duplicate rules and more specific rules that are covered by more general rules within the rule base. From this last definition and the membership function fusion method presented in [39], we propose a simple and efficient methodology (see experimental results) that searches rules that cover contiguous areas of their input variables associated with the same state of the output variable, and recently, it builds a new and more general rule that covers the contiguous states of the input variables and the same state of the output variable. These original rules will be redundant with the new rule, and then they can be substituted by this new more general rule.

To perform this task, we can apply an algorithm composed of the following steps:

- It searches for rules with overlap in the antecedent and consequent.
- It makes groups of rules with only one difference in the same proposition of the antecedent.
- For each of these groups, it selects the rules with adjacent fuzzy sets in this proposition. These redundant rules can be simplified into only one rule.

The new rule will be the same as the old rules except in their different proposition, and now, their new fuzzy set will be built by the composition of the involved adjacent fuzzy sets.

#### 3.2.3. Algorithm Proposed to Evaluate the Decrease in Performance

A reduction of rules can produce a lack of accuracy in modeling or control problems. Therefore, it is important to evaluate the utility of a rule by analyzing its impact on the global system behavior. To evaluate the accuracy of a new KB, this paper proposes the use of an algorithm that contains the following steps:

- Calculate the “Absolute Errors” for each element of a wide set of states in the space of “n” input variables, as follows:
  (1) $$\mathrm{AE}=\left(\left|{\mathrm{output}}_{\mathrm{system}\;1}-{\mathrm{output}}_{\mathrm{system}\;2}\right|\right)$$
  in which each individual error has been obtained by subtracting two FRBS outputs (output_FRBSystem 1_ – output_FRBSystem 2_).
- Calculate the “Sum of the Absolute Errors” (SAE), which must be obtained by considering a wide set of states in the space of “n” input variables.
  (2) $$\mathrm{SAE}=\sum_{i_{1}=1}^{L_{1}}\cdots \sum_{i_{j}=1}^{L_{j}}\cdots \sum_{i_{n}=1}^{L_{n}}\left(\left|{\text{output}}_{\text{FRBSystem}\;1}-{\text{output}}_{\text{FRBSystem}\;2}\right|\right)$$
  where L_1_, L_j_, L_n_ point to the higher value of the space of the input variables first, jth and nth. According to this definition, this parameter reports the global difference between the outputs of two FRBSs.
- Calculation of the “Relative Error” (RE), as follows:
  (3) $$\mathrm{RE}=\frac{\mathrm{SAE}}{\sum_{i_{1}=1}^{L_{1}}\cdots \sum_{i_{j}=1}^{L_{j}}\cdots \sum_{i_{n}=1}^{L_{n}}\left({\text{output}}_{\text{FRBSystem}\;1}\right)}$$

According to this definition, the RE parameter generates a value in the interval (0,1) that has been obtained by normalizing the SAE with the addition of FRBS 1 outputs. If at least one of these indicators is higher than its threshold of admissible value, we can infer that the KB used in system 2 may not have completeness or consistency.

#### 3.2.4. Transformation of the Base of Rules descriptive into approximates

To make the most out of approximate and descriptive FRBS advantages, this approach enables the use of descriptive and approximate KBs, while the inference engine of the embedded FRBS works as an approximate Mamdani-type.

If a descriptive KB composition is chosen, the human expert must specify the linguistic labels associated with each linguistic variable, the descriptive fuzzy sets defining the semantic of each label and the linguistic rules composing the RB. In this case, there is a simple transformation, in which each rule substitutes the descriptive fuzzy sets by approximate sets and is required to construct an equivalent approximate KB.

Therefore, in this process, each descriptive fuzzy set that points to a specific fuzzy set of a linguistic partition of the underlying linguistic variable is substituted by an approximate fuzzy set that contains the numeric definition of the membership function of the specific fuzzy set associated with each variable.

#### 3.2.5. Communications Protocol

Later, when the descriptive KB is transformed into an approximate KB, a communications protocol is used to transmit the KBs. As described in [47], this application layer protocol has been implemented on Sun SPOTs [48] and on a computer and it has been designed to allow the distribution of entire or partial KBs (including variables and fuzzy sets) to the sensor nodes, to provide the collection of inferred data, to manage the sensor nodes, and to integrate with other measurement applications.

This protocol has encouraged the versatility of its services to make sensors capable of achieving a wide range of tasks, which was one of the conclusions shown in [49]. According to [50], the main purpose of this protocol is to provide an Application Service Interface (ASI) to manage the problems generated with applications that access every low-level system of the device.

The advantages associated with the use of the wireless intelligent sensor management application protocol presented can be summarized with:

- A service interface that is independent of the platform.
- To provide support to a complete or incremental update of KBs.
- Versatility in the way in which sensor nodes notify their measurements, results or inferences.
- To provide support to minimize communication and processing and to optimize battery usage.
- To provide support to new types of probes, actuators or sensors due to the open data format and hierarchical labeling system used.

## 4. Experimental Results

To illustrate the proposed methodology of rule reduction and the evaluation of performances, three experiments have been performed to model, with simple KBs, the behavior of two plagues of the olive tree: prays (*Prays oleae* Bern.) and repilo (*Spilocaea oleagina*). The life cycles of these plagues depend on the humidity, temperature and photoperiod, but they are not the same [51]. The common steps of each experiment consist of the following:

- KB generation in the visual interface.
- Rule transformations.
- KB transmission.
- Modeling a wide set of system states (400,000) in an FRBS.
- Run the modeling process until achieving a 5% discharge in the battery, and calculating the number of necessary inferences.
- Transmission of results.

**Figure 3.**
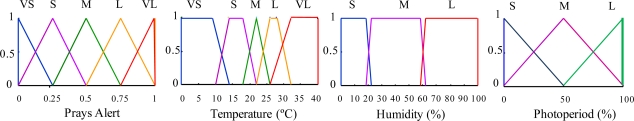
Membership functions of the input and output variable fuzzy sets defined in specific approximate rules for prays modeling.

**Figure 4.**
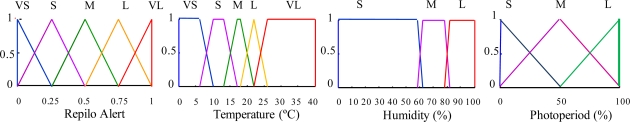
Membership functions of the inputs and output variable fuzzy sets defined in specific approximate rules for repilo modeling.

Each experiment has a specific role. In Experiment 1, an approximate KB is composed of an RB with two groups of rules (GR) (Tables 1 and 2) and their associated specific fuzzy set (FS) definitions (Figures 3 and 4).

**Figure 5.**
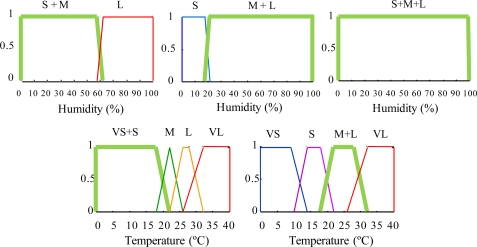
Input fuzzy sets for prays modeling, in reduced KB.

**Figure 6.**
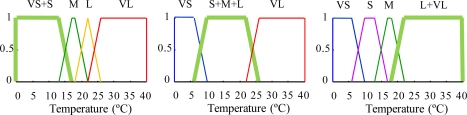
Input fuzzy sets for repilo modeling, in reduced KB.

In Experiment 2, an approximate KB is composed of a RB with two GR (Tables 3 and 4) and the associated specific FS definitions (Figures 5 and 6). In this case, the rules and FS definitions have been obtained using the redundancy reduction module. As can be observed, this algorithm decreases twelve rules for prays RB and eleven rules for the repilo KB (Tables 3 and 4) and generates, by means of fusion, new FSs (six for prays and four for repilo) (Figures 5 and 6).

With the purpose of illustrating the rule reduction process, an example of reduction is shown. In the GR used to model prays (Table 1), there is a set of three rules with the same consequent and the same second and third proposition in their antecedents:

R1: If Humidity is S and Temperature is VS and Photoperiod is S Then Prays Alert is VS
R2: If Humidity is M and Temperature is VS and Photoperiod is S Then Prays Alert is VS
R3: If Humidity is L and Temperature is VS and Photoperiod is S Then Prays Alert is VS

Applying the proposed algorithm, these original rules can be simplified into a single rule (New R1).

New R1: If Humidity is S&M&L and Temperature is VS and Photoperiod is S Then Prays Alert is VS

Except for the first proposition in the antecedent, the rule “New R1” is equal to the three originals. Now, the FS associated with the “Humidity” variable is “S&M&L” (Figure 5), which has been obtained by means of composition of the three original FS (Figure 3).

**Figure 7.**
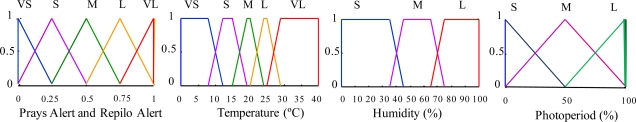
Membership functions of the input and output variable fuzzy sets included in common descriptive KB for prays and repilo modeling.

In Experiment 3, a descriptive KB is composed of an RB with two GRs (Tables 1 and 2) and the definition of linguistic labels associated with each linguistic variable (Figure 7). In this case, the use of linguistic labels prevents specific FS definitions for each GR.

To evaluate the accuracy of the KBs used in the second and third experiments, we calculate the parameters AE, SAE and RE, which are explained in Section 3.2.3. In this case, the calculation of these parameters has been performed by taking into account that System 1 is an FRBS with the original approximate KB and System 2 is an FRBS with the following:

- A descriptive KB, if we are considering the second experiment.
- A reduced approximate KB, if we are considering the third experiment.

To show the results of this evaluation, we have obtained the following indicators:

(a) The “Sum of the Absolute Errors” (SAE) and the “Relative Error” (RE) have been calculated considering a wide set of system states (400,000) in the space of the photoperiod, temperature and humidity variables.

To calculate these parameters, we have used Equations (2) and (3), taking into account that we now have three input variables: photoperiod, temperature and humidity and L_1_ = 100, L_2_ = 40, L_3_ = 100.

Considering that the proposed FRBS is used to model the behavior of two plagues (prays and repilo) of the olive tree, their global SAE must be calculated as the sum of the SAEs for prays and repilo.

(4) $${\text{SAE}}_{\text{Global}}={\text{SAE}}_{\text{Prays}}+{\text{SAE}}_{\text{Repilo}}$$

In the same way, the global RE must be calculated as the average of the REs for prays and repilo.

(5) $${\mathrm{RE}}_{\mathrm{Global}}=\frac{\left({\mathrm{RE}}_{\mathrm{Prays}}+{\mathrm{RE}}_{\mathrm{Repilo}}\right)}{2}$$

In Tables 5 and 6, we can see the values taken by these accuracy parameters.

As can be observed in Table 6, in the case of modeling with a reduced approximate KB, the parameters RE_Glogal_ and SAE_Global_ take low values (relatively close to zero), mainly if they are compared with their values in the case of modeling with a descriptive KB (see Table 5), so much so that they are more than fifty nine times greater. Therefore, the use of the reduced approximate KB entails a small decrease in the accuracy of the modeling process, while the use of the descriptive KB entails an unacceptable degradation of the accuracy.

(b). In each of the second and third experiments, we calculated 400,000 values of the AE parameter. Because this number is too large to be shown in a table, to be informative about the values of this parameter, we present the following:

First is the evolution of the sum of the AE for each state of a wide set of values (100) in the space of the photoperiod variable:

1. In the case of modeling prays.
2. In the case of modeling repilo.

Second, for some values of the photoperiod variable, the evolution of the AE for a wide set of states (4,000) in the space of temperature and humidity variables are shown:

1. In the case of modeling prays.
2. In the case of modeling repilo.

**Figure 8.**
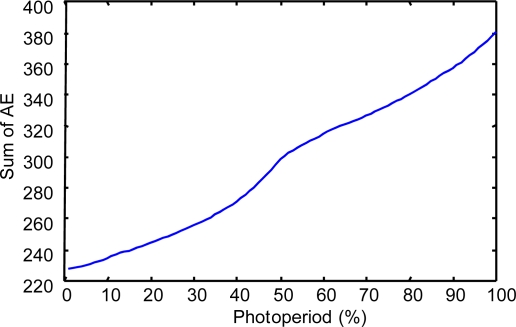
The sum of the AE for different values of the photoperiod variable, in the case of modeling prays with a descriptive KB.

**Figure 9.**
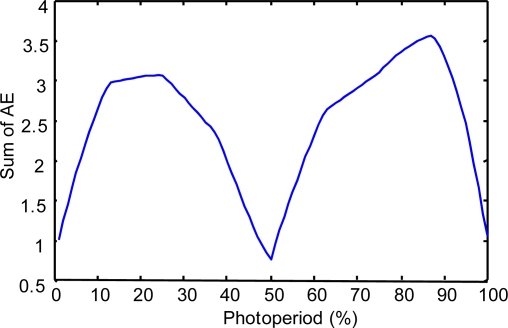
The sum of the AE for different values of the photoperiod variable, in the case of modeling prays with a reduced approximate KB.

**Figure 10.**
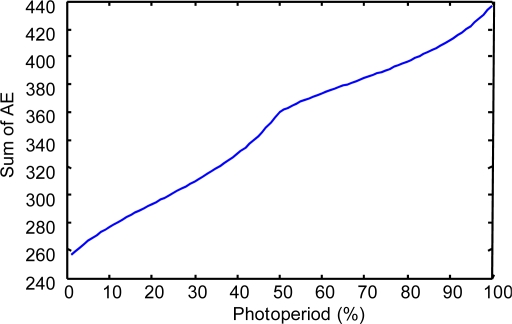
The sum of the AE for different values of the photoperiod variable, in the case of modeling repilo with a descriptive KB.

**Figure 11.**
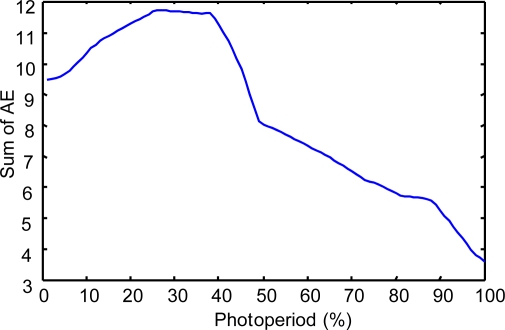
The sum of the AE for different values of the photoperiod variable, in the case of modeling repilo with a reduced approximate KB.

**Figure 12.**
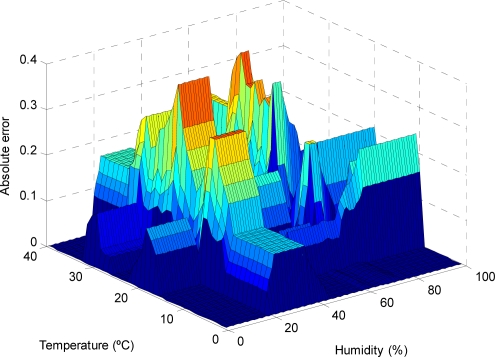
The AE for different values of the temperature and humidity variables (considering photoperiod = 80%), in the case of modeling prays with a descriptive KB.

**Figure 13.**
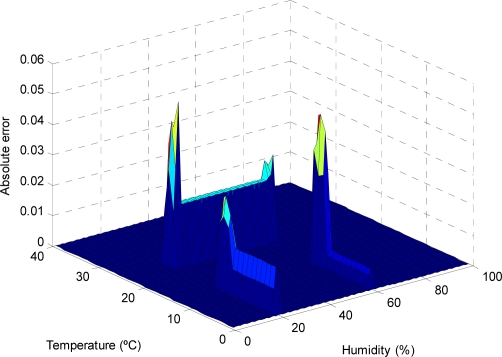
The AE for different values of the temperature and humidity variables (considering photoperiod = 80%), in the case of modeling prays with a reduced approximate KB.

**Figure 14.**
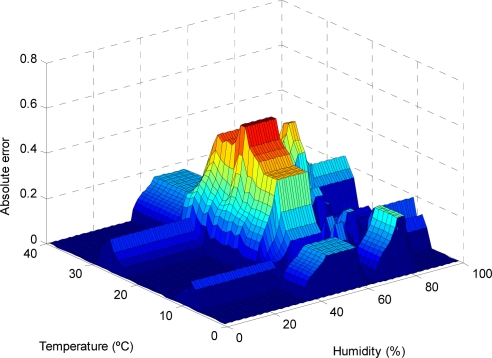
The AE for different values of the temperature and humidity variables (considering photoperiod = 80%), in the case of modeling with a descriptive KB.

**Figure 15.**
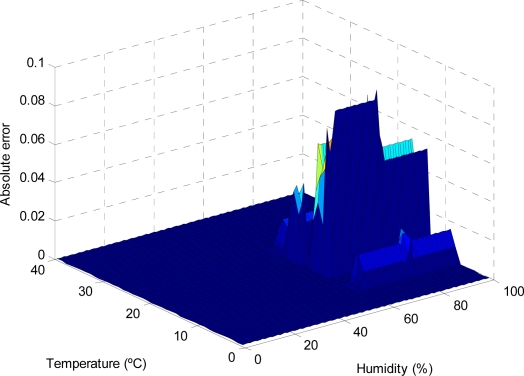
The AE for different values of the temperature and humidity variables (considering photoperiod = 20%), in the case of modeling with a reduced approximate KB.

As can be observed in the previous figures, the use of the reduced approximate KB proposed entails a small decrease in the accuracy, which is limited to small regions of the input variable space (see Figures 9, 11, 13 and 15); otherwise, the use of the descriptive KB proposed entails a highest decrease in the accuracy that is widespread on extensive regions of the input variable space (see Figures 8, 10, 12 and 14). To evaluate the effect on battery consumption, two sets of measurements have been performed over each one of the FRBS proposed in the three experiments:

- The run time necessary to execute 400,000 continued inferences. The results obtained (Table 7) show that the use of the approximate reduced KB versus the original KB saves approximately 25.64% in battery consumption. These rule reductions have been obtained without an appreciable lack of accuracy in modeling (RE = 0.47%).
- The number of inferences and the battery consumption necessary to obtain a 5% discharge in the battery.

The results obtained (Table 8) show that the use of the approximate reduced KB versus the original KB makes it possible to have an increase of 50% in the number of inferences.

From the analysis of the experimental results obtained, we notice that it has been possible to compose an optimized KB, adapted to model olive plagues, which generates a decrease in the FRBS run time (an increase in the inference rate) and a decrease in battery consumption (a decrease in the time of discharge in the battery sensor), without substantially decreasing the accuracy.

## 5. Conclusions

This work has presented a distributed architecture proposal for collaborative FRBSs embedded in WSNs, which has been designed to optimize the implementation of IS by means of WSNs. This architecture includes the following:

- A design of the inference engine adapted to achieve a high level of accuracy by means of the use of an approximate KB.
- A visual interface to facilitate the generation of descriptive KBs with a large degree of interpretability in the linguistic rules.
- A module to decrease the redundancy and the complexity of the KB without a substantial decrease in their completeness and consistency.
- A module to evaluate the accuracy of the new reduced KB that is obtained.
- A module that makes possible the transformation of descriptive KBs into approximate KBs, for the purpose of achieving a high level of accuracy beginning with rules that have a high degree of interpretability.
- A communications protocol for the distribution of KBs, data and measures.

This architecture, the proposed methodologies of rule reduction and the evaluation of performances have been applied to the problem of modeling two plagues of the olive tree: the Prays and the Repilo.

The results have shown the effectiveness of the proposed structure for optimizing the execution of an FRBS embedded into a sensor. In this sense, we can observe that the use of a reduced approximate KB, obtained according to the proposed architecture, entails the following:

- A small decrease in the accuracy of the modeling process, and this decrease is limited to small regions of the input variable space.
- A substantial decrease in FRBS run time (increasing the inference rate) and in battery consumption (decreasing the time for discharge of the sensor battery).

These results suggest that the architecture presented in this paper allows a decrease in the consumption of the resources required (memory, CPU and battery) by an FRBS embedded in a node of a WSN, without a substantial decrease in the accuracy of the inferred values.

## Figures and Tables

**Table 1.** Group of rules used to model prays (in specific and common KB).

***Group of rules used in the model of “Prays” with Short Photoperiod***						
***Prays Alert***	***Temperature***					
***Humidity***		**VS**	**S**	**M**	**L**	**VL**
	**S**	**VS**	**VS**	**S**	**S**	**VS**
	**M**	**VS**	**VS**	**M**	**S**	**VS**
	**L**	**VS**	**M**	**L**	**M**	**S**

***Group of rules used in the model of “Prays” with Medium Photoperiod***						
***Prays Alert***	***Temperature***					
***Humidity***		**VS**	**S**	**M**	**L**	**VL**
	**S**	**VS**	**VS**	**S**	**S**	**VS**
	**M**	**VS**	**S**	**M**	**M**	**S**
	**L**	**VS**	**M**	**VL**	**L**	**M**

***Group of rules used in the model of “Prays” with Long Photoperiod***						
***Prays Alert***	***Temperature***					
***Humidity***		**VS**	**S**	**M**	**L**	**VL**
	**S**	**VS**	**VS**	**S**	**S**	**VS**
	**M**	**S**	**M**	**L**	**M**	**S**
	**L**	**S**	**L**	**VL**	**L**	**M**

**Table 2.** Group of rules used to model repilo (in specific and common KB).

***Group of rules used in the model of “Repilo” with Short Photoperiod***						
***Repilo Alert***	***Temperature***					
***Humidity***		**VS**	**S**	**M**	**L**	**VL**
	**S**	**VS**	**S**	**S**	**S**	**VS**
	**M**	**S**	**S**	**M**	**S**	**S**
	**L**	**M**	**M**	**L**	**M**	**M**

***Group of rules used in the model of “Repilo” with Medium Photoperiod***						
***Repilo Alert***	***Temperature***					
***Humidity***		**VS**	**S**	**M**	**L**	**VL**
	**S**	**VS**	**S**	**S**	**S**	**VS**
	**M**	**S**	**M**	**L**	**M**	**S**
	**L**	**M**	**L**	**VL**	**L**	**M**

***Group of rules used in the model of “Repilo” with Long Photoperiod***						
***Repilo Alert***	***Temperature***					
***Humidity***		**VS**	**S**	**M**	**L**	**VL**
	**S**	**VS**	**S**	**S**	**S**	**VS**
	**M**	**S**	**L**	**VL**	**L**	**S**
	**L**	**M**	**VL**	**VL**	**VL**	**M**

**Table 3.** Reduced group of rules used to model prays.

***Group of rules used in the model of “Prays” with Short Photoperiod***						
***Prays Alert***	***Temperature***					
***Humidity***		**VS**	**S**	**M**	**L**	**VL**
	**S**	**VS**	**VS**	**S**	**S**	**VS**
	**M**			**M**		
	**L**		**M**	**L**	**M**	**S**

***Group of rules used in the model of “Prays” with Medium Photoperiod***						
***Prays Alert***	***Temperature***					
***Humidity***		**VS**	**S**	**M**	**L**	**VL**
	**S**	**VS**	**VS**	**S**		**VS**
	**M**		**S**	**M**		**S**
	**L**		**M**	**VL**	**L**	**M**

***Group of rules used in the model of “Prays” with Long Photoperiod***						
***Prays Alert***	***Temperature***					
***Humidity***		**VS**	**S**	**M**	**L**	**VL**
	**S**	**VS**		**S**		**VS**
	**M**	**S**	**M**	**L**	**M**	**S**
	**L**		**L**	**VL**	**L**	**M**

**Table 4.** Reduced group of rules used to model repilo.

***Group of rules used in the model of “Repilo” with Short Photoperiod***						
***Repilo Alert***	***Temperature***					
***Humidity***		**VS**	**S**	**M**	**L**	**VL**
	**S**	**VS**	**S**			**VS**
	**M**	**S**		**M**	**S**	
	**L**	**M**		**L**	**M**	

***Group of rules used in the model of “Repilo” with Medium Photoperiod***						
***Repilo Alert***	***Temperature***					
***Humidity***		**VS**	**S**	**M**	**L**	**VL**
	**S**	**VS**	**S**			**VS**
	**M**	**S**	**M**	**L**	**M**	**S**
	**L**	**M**	**L**	**VL**	**L**	**M**

***Group of rules used in the model of “Repilo” with Long Photoperiod***						
***Repilo Alert***	***Temperature***					
***Humidity***		**VS**	**S**	**M**	**L**	**VL**
	**S**	**VS**	**S**			**VS**
	**M**	**S**	**L**	**VL**	**L**	**S**
	**L**	**M**	**VL**			**M**

**Table 5.** Accuracy parameters in the case of modeling with a descriptive KB.

	**Prays**	**Repilo**	**Global**
**SAE**	29,110.95	34,460.26	63,571.21
**RE (%)**	25.09	31.11	28.1

**Table 6.** Accuracy parameters in the case of modeling with a reduced approximate KB.

	**Prays**	**Repilo**	**Global**
**SAE**	246.97	828.25	1,075.22
**RE (%)**	0.2	0.75	0.475

**Table 7.** Comparison of performance considering different types of embedded FRBS.

	Execution of 400,000 continued inferences				
FBRS	Time (ms)	Increase (%)	Consumption (mA)	Increase (%)	RE (%)
Approximate	64,455	0.00	1.95	0.00	0.00
Descriptive	63,376	−1.67	1.92	−1.54	28.10
Approximate Reduced	47,549	−26.23	1.45	−25.64	0.47

**Table 8.** Comparison of the number of necessary inferences to produce a 5% discharge in battery.

	Number of necessary inferences to produce a 5% discharge in battery			
FBRS	Number of inferences	Battery Consumption (mA)	Increase in number of inferences (%)	Number of rules
Approximate	7,200,000	33.69	0.00	90
Descriptive	8,000,000	32.20	11.1	90
Approximate Reduced	10,800,000	33.29	50	66
